# Psychopharmacology In Myasthenia Gravis Patients: A Case Study

**DOI:** 10.1192/j.eurpsy.2023.622

**Published:** 2023-07-19

**Authors:** T. M. Afonso, F. Ferreira, C. Cativo, S. Martins, A. Ramos, M. Pinto

**Affiliations:** Mental Health Department, Hospital Professor Doutor Fernando Fonseca, Amadora, Portugal

## Abstract

**Introduction:**

Myasthenia gravis (MG) is an autoimmune disease that affects the neuromuscular junction. It causes generalized muscle weakness that may include the respiratory muscles, potentially leading to a medical emergency known as a myasthenic crisis. Several medications, including some antipsychotics, have been shown to worsen myasthenia gravis symptoms.

**Objectives:**

We aim to summarize the current knowledge on the use of psychopharmacological treatments in patients with MG.

**Methods:**

Non-systematic review of the literature was performed in PubMed/Medscape database. Case report of a patient who was admitted and treated in our inward patient unit.

**Results:**

We present a clinical case of a 64-year-old man diagnosed with Bipolar Disease at the age of 18 and recently diagnosed with MG (he was hospitalized in Neurology Department, pyridostigmine was introduced and lithium was reduced to half dose). Three months later he was admitted to the emergency department due to behavior and speech disorganization, persecutory delusional ideas, insomnia and caregiver exhaustion. During his hospitalization lithium was increased to 1200 mg. At day 8 of admission the patient started to show weakness of neck extensor muscles, due to that he was evaluated by neurology, lithium was stopped and haloperidol was increased resulting in clinical improvement.

**Conclusions:**

Psychotropic choice in patients with MG can be challenging due to their anticholinergic properties that can exacerbate MG symptoms with potential deterioration to a myasthenic crisis. There is a great need for evidence-based data on the safety and efficacy of psychotropic medications in MG.

**Disclosure of Interest:**

None Declared

